# Calcitriol reduces eosinophil cytolysis and release of cytotoxic granules *in vitro*

**DOI:** 10.1186/1710-1492-10-S1-A3

**Published:** 2014-03-03

**Authors:** Caroline Ethier, Yingqi Wu, Paige Lacy, Lisa Cameron, Francis Davoine

**Affiliations:** 1 Pulmonary Research Group - Department of Medicine, Edmonton, Alberta, Canada; 2 Campus Saint-Jean, University of Alberta, Edmonton, Alberta, Canada, T6G 1A6

## Background

Epidemiological studies show a strong correlation between vitamin D deficiency and asthma severity. Genetic studies have identified vitamin D receptor polymorphisms as a risk factor for asthma in diverse human populations. Calcitriol (1,25-dihydroxyvitamin D3), the physiologically active metabolite of mammalian vitamin D, is able to modulate receptor and cytokine expression as well as cell differentiation and maturation of various leukocytes in an anti-inflammatory manner. Despite the role of eosinophils in allergic asthma pathology, little is known about the effects of calcitriol on eosinophil biology. Calcitriol has a direct modulatory effect on eosinophil survival and effector functions *in vitro*.

## Methods

Human peripheral blood eosinophils from atopic donors were isolated and incubated with calcitriol (VD) and interleukin-5 (IL-5). Dose-response assays tested physiological doses of calcitriol (0.01-100 nM). The potentiating effect of calcitriol (10 nM) on eosinophil survival with IL-5 (1 ng/mL) was investigated on a 14-day time course. Viability/apoptosis/necrosis levels were obtained using an Annexin-V/PI flow cytometry assay. Eosinophil crystalloid granules and EPX release were measured by CD63 and EPX monoclonal antibody staining using flow cytometry. EPX activity was determined using an OPD substrate colorimetric assay.

## Results

**Figure 1:**
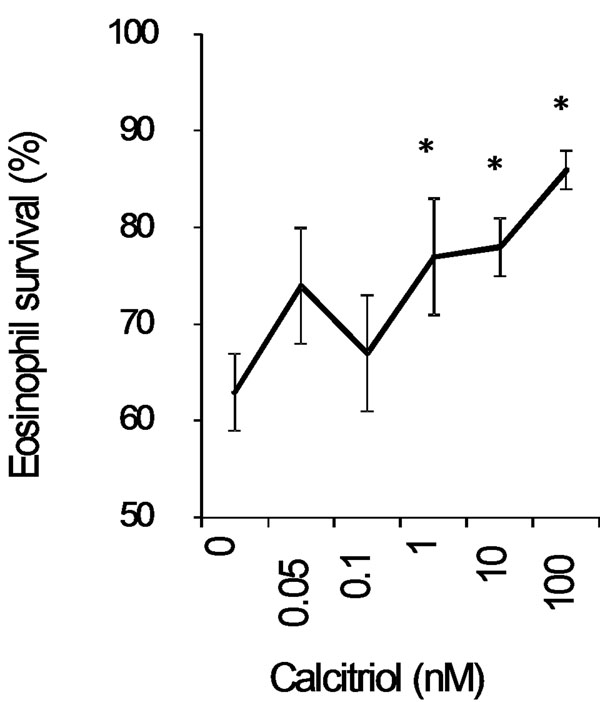


**Figure 2:**
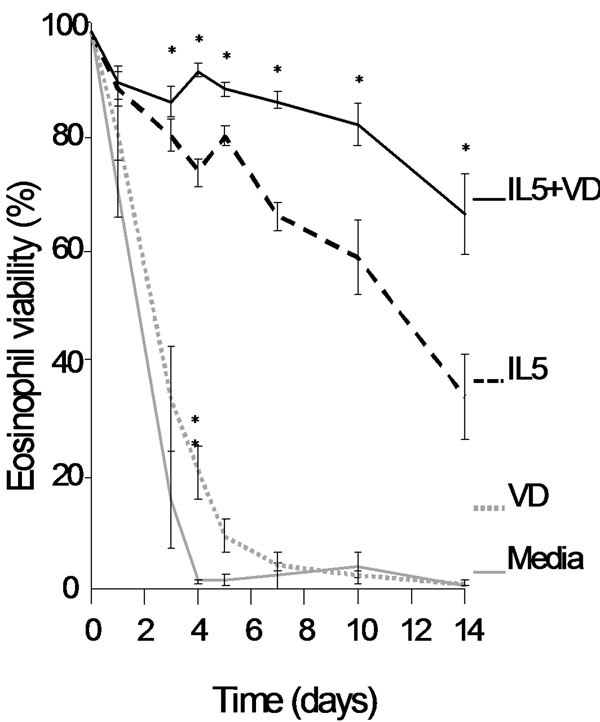


**Figure 3:**
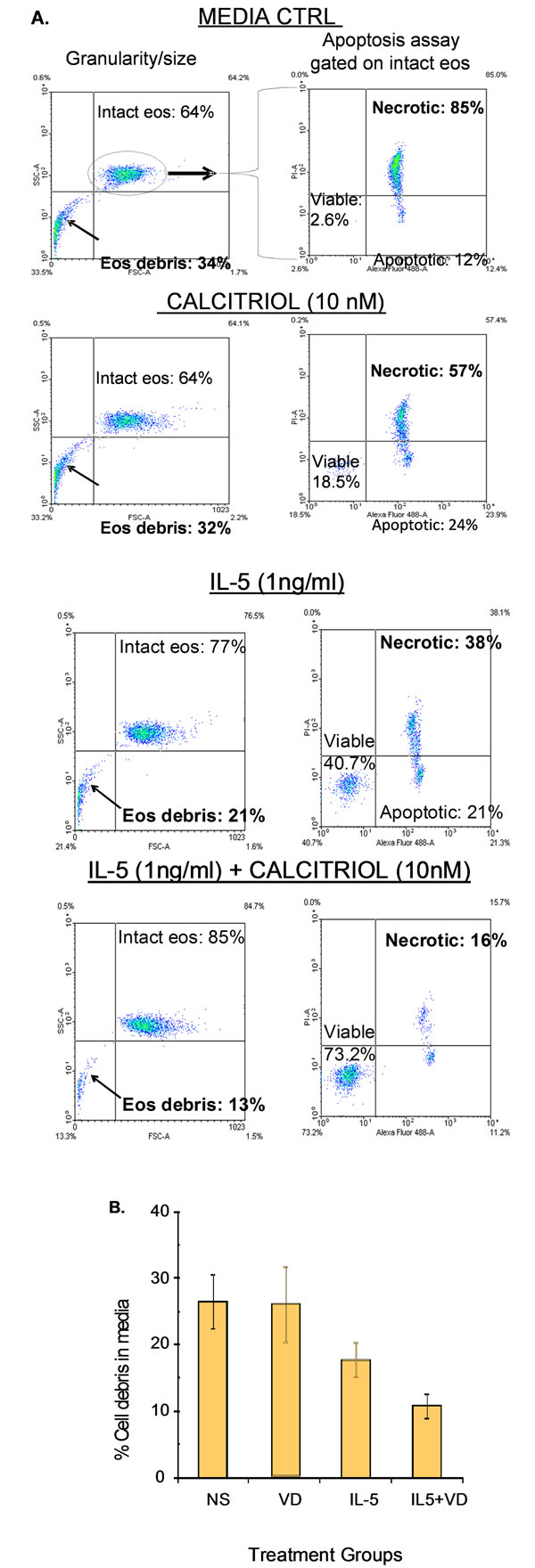


**Figure 4:**
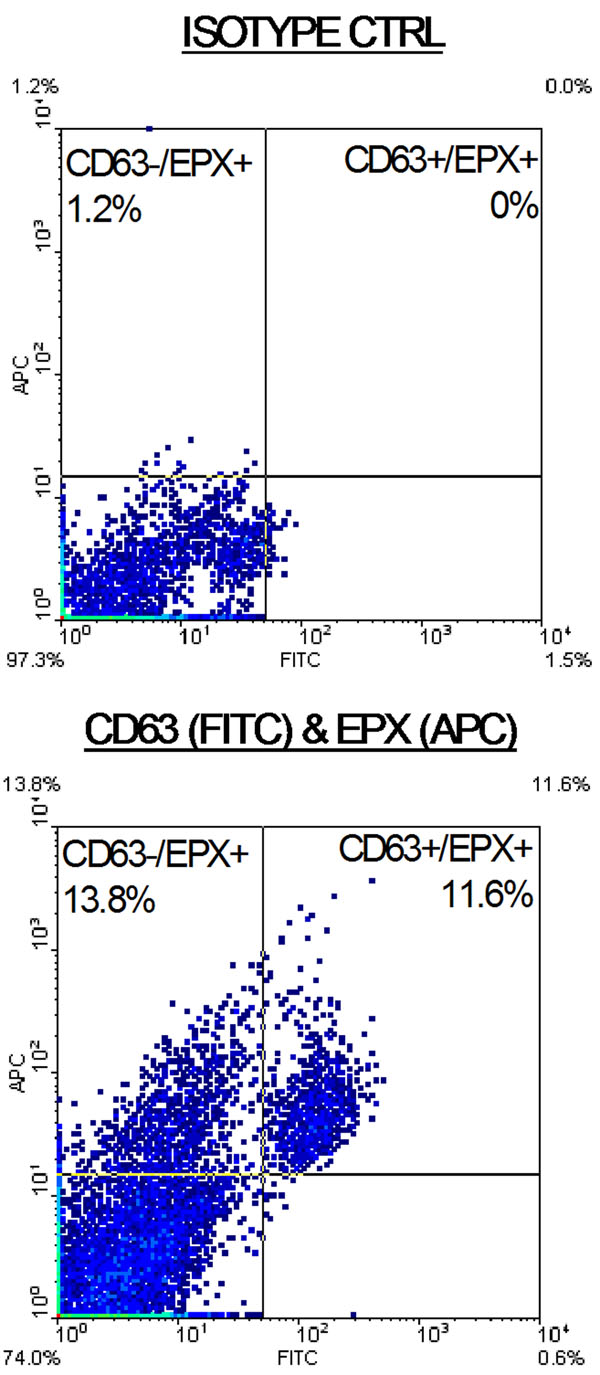


**Figure 5:**
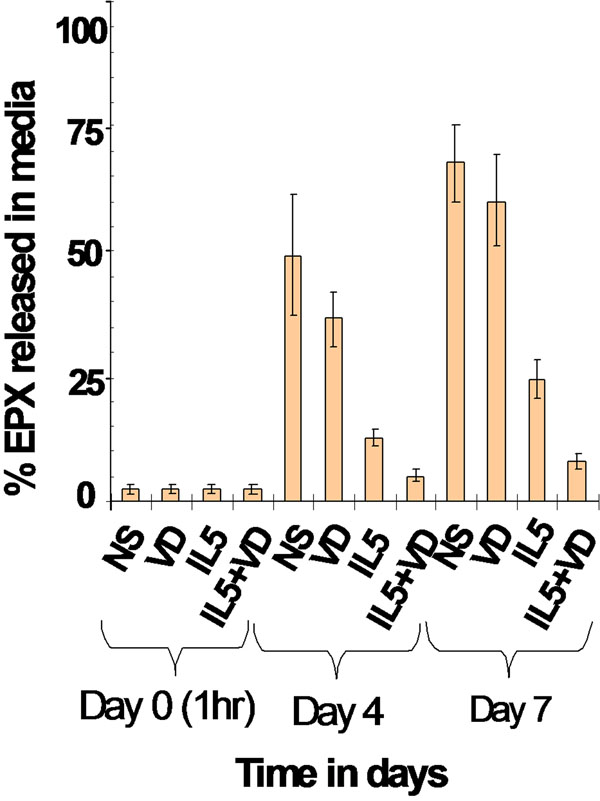


**Figure 6:**
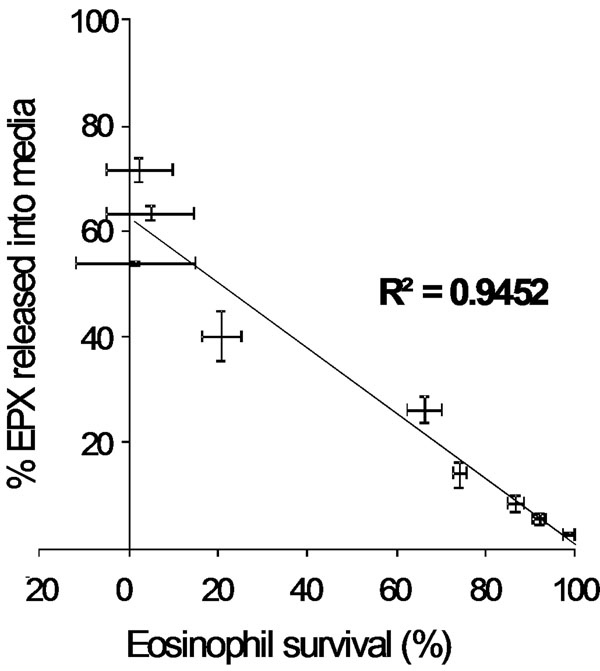


Eosinophil survival increased after 24 h of calcitriol treatment in a dose-dependent manner (Fig1. *n* = 5). Calcitriol alone yielded similar eosinophil survival rates (Fig2.4.5 ± 1.5%, *n* = 11) after 7 days compared to control media (Fig2. 2.1 ± 2.1%, *n* = 11). In contrast, calcitriol potentiated the survival effect of IL-5 starting from day 7. At 14 days, 66 ± 7% of eosinophils were still intact when treated with calcitriol and IL-5, compared to IL-5 alone (Fig2. 34 ± 8%, *p* < 0.05, *n* = 4). Cells treated with IL-5 alone showed increased necrosis from day 7 onward with 32% additional necrotic eosinophils at day 14, compared to the combined treatment. As cell debris increased in correlation with necrosis (Fig3 A&B.), low-SSC/FSC events stained positive for CD63 and EPX confirming whole, intact crystalloid granules along with free EPX in media (Fig4.). Moreover, EPX activity in media was reduced on days 4 and 7 following calcitriol and IL-5 treatment (Fig5.). Overall, reduced spontaneous EPX release strongly correlated with increased eosinophil survival (Fig6. *r^2^* = 0.96, *n* = 4-11).

## Conclusions

These findings support the hypothesis that calcitriol plays an anti-inflammatory role by decreasing cytotoxic granule release into airway mucosal tissues during allergic inflammatory responses, therefore reducing mucosal inflammation and tissue damage.

